# Self-Management Support Using a Digital Health System Compared With Usual Care for Chronic Obstructive Pulmonary Disease: Randomized Controlled Trial

**DOI:** 10.2196/jmir.7116

**Published:** 2017-05-03

**Authors:** Andrew Farmer, Veronika Williams, Carmelo Velardo, Syed Ahmar Shah, Ly-Mee Yu, Heather Rutter, Louise Jones, Nicola Williams, Carl Heneghan, Jonathan Price, Maxine Hardinge, Lionel Tarassenko

**Affiliations:** ^1^Nuffield Department of Primary Care Health SciencesUniversity of OxfordOxfordUnited Kingdom; ^2^Institute of Biomedical EngineeringDepartment of Engineering ScienceUniversity of OxfordOxfordUnited Kingdom; ^3^Department of PsychiatryUniversity of OxfordOxfordUnited Kingdom; ^4^Oxford University Hospitals NHS Foundation TrustOxfordUnited Kingdom

**Keywords:** pulmonary disease, chronic obstructive, telehealth, self-care, randomized controlled trial

## Abstract

**Background:**

We conducted a randomized controlled trial of a digital health system supporting clinical care through monitoring and self-management support in community-based patients with moderate to very severe chronic obstructive pulmonary disease (COPD).

**Objective:**

The aim of this study was to determine the efficacy of a fully automated Internet-linked, tablet computer-based system of monitoring and self-management support (EDGE‚ sElf-management anD support proGrammE) in improving quality of life and clinical outcomes.

**Methods:**

We compared daily use of EDGE with usual care for 12 months. The primary outcome was COPD-specific health status measured with the St George’s Respiratory Questionnaire for COPD (SGRQ-C).

**Results:**

A total of 166 patients were randomized (110 EDGE, 56 usual care). All patients were included in an intention to treat analysis. The estimated difference in SGRQ-C at 12 months (EDGE−usual care) was −1.7 with a 95% CI of −6.6 to 3.2 (*P*=.49). The relative risk of hospital admission for EDGE was 0.83 (0.56-1.24, *P*=.37) compared with usual care. Generic health status (EQ-5D, EuroQol 5-Dimension Questionnaire) between the groups differed significantly with better health status for the EDGE group (0.076, 95% CI 0.008-0.14, *P*=.03). The median number of visits to general practitioners for EDGE versus usual care were 4 versus 5.5 (*P*=.06) and to practice nurses were 1.5 versus 2.5 (*P*=.03), respectively.

**Conclusions:**

The EDGE clinical trial does not provide evidence for an effect on COPD-specific health status in comparison with usual care, despite uptake of the intervention. However, there appears to be an overall benefit in generic health status; and the effect sizes for improved depression score, reductions in hospital admissions, and general practice visits warrants further evaluation and could make an important contribution to supporting people with COPD.

**Trial registration:**

International Standard Randomized Controlled Trial Number (ISRCTN): 40367841; http://www.isrctn.com/ISRCTN40367841 (Archived by WebCite at http://www.webcitation.org/6pmfIJ9KK)

## Introduction

### Background

Chronic obstructive pulmonary disease (COPD) is a major cause of morbidity and mortality [1], with global costs of US $141 billion. In the United Kingdom, the total annual estimated cost of COPD to the National Health Service (NHS) is over £800 million, over half attributable to hospital-based care [2]. The impact of COPD on the health-related quality of life of patients is well-established [3,4].

Correct use of long-acting beta-agonists alone or in combination with anti-inflammatory inhaled steroids can reduce the overall rate of exacerbations by 20% [5]. Early recognition of and intervention during an exacerbation can also reduce admission risk [6]. Training and support for patients in the self-management of their condition, for example, through outpatient pulmonary rehabilitation [7], improves quality of life and can reduce unplanned hospital admissions [8-10]. There is an evidence base for use of psychological therapies such as cognitive behavioral therapy [11], although a wider range of interventions designed to promote self-management do not appear to work consistently, possibly because of nonadherence to the self-management program or to action plans put in place [12,13], or because not all the components required to improve self-management are included.

There is a need to identify different forms of self-management that can improve outcomes and to develop and optimize ways of delivering available interventions to maximize effectiveness and safety. Delivery of interventions at a widescale and continuing the intervention over time are likely to be important factors addressing feasibility and maintaining effectiveness. Use of converging computer and communication technologies in the form of digital health interventions offers a means of helping patients monitor their condition, providing support in interpreting data for self-management, and supplying a means of delivering individually tailored education and treatment plans.

### Digital Health Intervention

Digital health interventions in COPD are increasingly employed, and particularly health apps have been found to show potential in improving symptoms management through self-management support [14]. Current digital health interventions and apps include a range of components, and mainly differ in the combination of tools used (patient education materials, exercise support, self-care plans, remote monitoring of symptoms, and clinical parameters) and input from clinicians (interpretation of patient data and feedback to patient through phone calls, text chats, or video calls) [15,16].

Understanding the heterogeneous nature of COPD events with a variable time course of symptom onset and recovery is particularly important to underpin interventions that may work for individual patients [17].

Systematic reviews of digital health interventions till date in COPD provide evidence to support continuing research [18], but recent large-scale evaluations have not shown convincing evidence of their effectiveness [19,20]. Three large trials of telehealth solutions for COPD have identified little or no benefit [19,21,22]. Limitations of current systems have included low compliance rates with the technology resulting from having to engage with a dedicated telehealth box; lack of flexibility in the technical specification; artifacts in data collection leading to high rates of false alerts; and limited personalization and support for self-management in the telehealth solution. Therefore, solutions need to be straightforward and easy-to-use by patients, be straightforward to implement with low-cost widely available technology, and utilize individualized predictive algorithms to address the variability of the condition [23-25].

Evaluation of digital health interventions require multiple perspectives to be considered within the evaluation [26]. We have recently carried out a cohort study in which we have shown that a tablet computer-based system for supporting patients with COPD is acceptable to them and feasible to use [23,27].

We therefore set out to determine the efficacy of an Internet-linked, tablet computer-based system of monitoring, and self-management support (EDGE‚ sElf-management anD support proGrammE) in improving quality of life and clinical outcomes when used by patients with moderate to very severe COPD.

## Methods

### Trial Design

**Figure 1:**
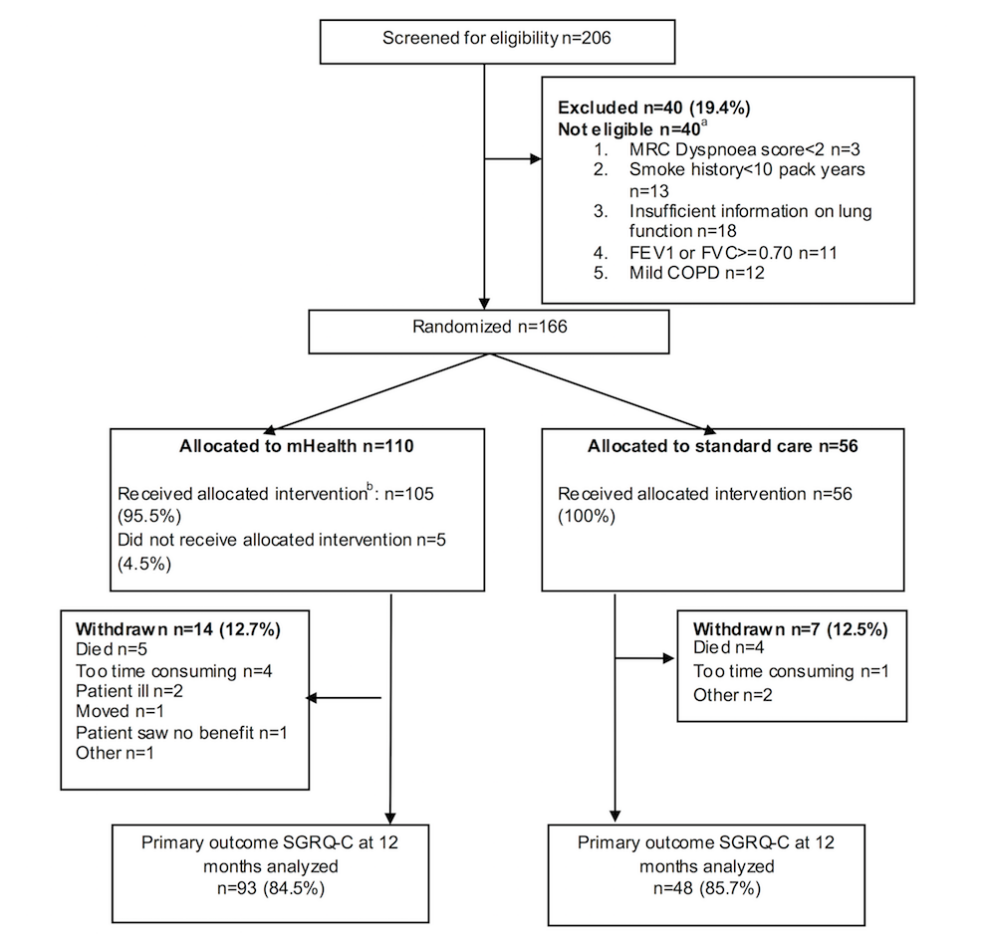
Participant flow legend. a: not mutually exclusive. b: defined as using the intervention for at least 30 days, during which time it was used for at least 3 out of 7 days per week. MRC: Medical Research Council; FEV1: forced expiratory volume in 1 s; FVC: forced vital capacity; COPD: chronic obstructive pulmonary disease, SGRQ-C: St George’s Respiratory Questionnaire for COPD.

EDGE for COPD is a multicenter, randomized controlled trial of 12-month duration [28]. Patients were individually randomized to receive either a system of care (the EDGE intervention) delivered via a digital health, Internet-linked platform implemented on a low-cost tablet computer (the EDGE platform) providing monitoring and self-management support or standardized usual care in a 2:1 allocation ratio (Figure 1). A 2:1 allocation ratio was chosen to maximize the information available about the use of the system across the population of individuals with COPD [29]. There were no changes to the design or methods of the study after recruitment commenced. An embedded qualitative study was used to carry out a process evaluation and to explore the experience of using the system.

### Participants

#### Eligibility Criteria for Participants

Eligible patients were aged ≥40 years with a confirmed diagnosis of COPD defined as a forced expiratory volume in 1 s (FEV1), post-bronchodilation of <70% [2], and a predicted ratio of FEV1 to forced vital capacity of <0.70. Eligible patients had a smoking-pack history >10 pack-years and a Medical Research Council dyspnea score of ≥2. Further trial eligibility criteria are reported in the trial protocol [28].

### Setting

Patients were recruited from a variety of settings encompassing primary and secondary care as well as community services. Patients attending respiratory hospital outpatient clinics and pulmonary rehabilitation courses in the adjacent counties of Oxfordshire and Berkshire, UK, were invited to participate. In addition, eligible patients were identified from primary care clinics and from those recently (within the preceding 2 weeks) discharged from hospital following a COPD-related admission.

### Trial Interventions

#### Intervention Development and Specification

On the basis of open-source app software, the EDGE platform was designed to be integrated in clinical care, by a team of clinicians and engineers working with patients. The platform was refined in a 6-month cohort study of a group of patients with COPD, who were selected using eligibility criteria matching those of the trial [27].

The EDGE intervention was designed to include tools to help patients identify exacerbations and to monitor their condition; to help support good compliance with inhaled medication; and to support psychological well-being. It incorporates a daily symptom diary consisting of standard questions about symptoms based on previous trial protocols [30,31]. Questions include general well-being, cough, breathlessness, sputum (quantity produced and color), and use of medications. A 30-s period of data acquisition using a Bluetooth-enabled pulse oximeter with finger probe (Nonin, PureSAT, 9560BT) manufactured by Nonin Medical Inc of Plymouth, Minnesota, USA, allows daily collection of heart rate and oxygen saturation data. Mood screening questionnaires [32-34] were presented each month for completion; further details are given in Multimedia Appendix 1.

The EDGE platform also includes a number of software modules, including videos tailored to the patient’s entries in the symptom diary or answers to the mood-screening questionnaires. These videos provide additional self-management support, and are listed in Multimedia Appendix 1. These include inhaler techniques, pulmonary rehabilitation exercises, and self-management techniques for breathlessness. All of the multimedia components were developed by the local clinical respiratory team and are based on current pulmonary rehabilitation interventions, for which there is good evidence of effectiveness in improving quality of life for patients with COPD [35]. As soon as the patient finishes using the app, data is securely transmitted to a server hosted behind NHS firewalls.

#### Use of the EDGE Platform

Participants allocated to receive the EDGE platform-based intervention were provided with an Android tablet computer (Samsung Galaxy Tab) running the app software and a Bluetooth-enabled oximeter probe.

Participants were briefly instructed on the use of the EDGE platform by the research nurse and given a brief information booklet detailing its use. Patients were informed that the EDGE platform was not a replacement for their usual clinical care, and that in the event of deterioration in their health they should contact their general practitioner or community respiratory nurse as usual. The intention of this approach was to establish the safe use of new technology, whereas not intervening in a way that might expose individuals to unintended harms [36].

In an initial 6-week period of use, EDGE users completed the symptom diary and recorded their oxygen saturation and heart rate with the pulse oximeter on a daily basis. Following this initial run-in period, the distributions of values for the oxygen saturation, heart rate, and symptom scores were calculated for the run-in period for each participant. The 97th centile was computed for the distribution of heart rate and symptom score, and the 3rd centile for the distribution of oxygen saturation; these were then used as the threshold for the participant safety alert for that parameter. The option to modify thresholds following a hospital admission was available. Participants continued to input their symptom data and clinical recordings daily throughout the duration of the trial.

One of three respiratory clinicians (nurse, physiotherapist, or doctor) reviewed a summary of the oxygen saturation, heart rate, and symptom diary module data twice weekly to ensure that data transmission was taking place and to deal with safety alerts. Data were assigned priority according to the number of “alerts” in the most recent two weeks since the last review. In this context, alerts were generated when the vital sign value (pulse rate or oxygen saturation) went above (or below) the safety threshold or the overall symptom score went above the safety threshold.

If data were not received or there were safety alerts, the participant record was accessed for review. If, on reviewing the data, there was judged to be a clinically important change in the data, then the patient was contacted either via message or telephone. A clinically important parameter was defined as either heart rate or symptom score moving above the 97th centile or oxygen saturation falling below the 3rd centile, as defined by baseline observation and persisting for at least 2 days. If depression or anxiety scores equaled or exceeded a threshold of 10, then the patient’s general practitioner was informed by letter. The intervention is summarized in Multimedia Appendix 2.

#### The Standardized Usual Care Intervention

Participants allocated to receive standardized usual care were provided with all the information given to those allocated to use the EDGE system, but without the use of a tablet computer or the facility for daily monitoring of symptoms and physiological variables. Participants were provided with leaflets based on those currently produced by the Oxfordshire Community Respiratory service. Further details are given in Multimedia Appendix 3.

#### Primary Outcome Measure

The primary outcome was the change in St George’s Respiratory Questionnaire for COPD (SGRQ-C) [37], which was used to assess COPD-specific health status from baseline to 1 year in patients with moderate to very severe COPD.

#### Secondary Outcome Measures

The following secondary endpoints were used to evaluate the impact of the intervention in comparison with usual care: (1) impact on hospital admissions (number of admissions and days in hospital) and deaths; (2) the number of recorded exacerbations defined as episodes in which antibiotics or oral steroids were prescribed or in which the patients were seen in the accident and emergency department or admitted to hospital in the presence of an acute change in respiratory symptoms defined as the presence of at least two symptoms, one of which should be major (major symptoms: change in sputum, more breathless, chest tight; minor symptom: unwell, tired, temperature, a cold) or a report of a patient taking more salbutamol, either blue inhaler or by nebulizer, for at least 48 h (Multimedia Appendix 1); (3) time to first exacerbation; (4) beliefs about respiratory medicine use measured with the Beliefs about Medicines Questionnaire [38]; (5) self-reported medication use measured with the Medication Adherence Report Schedule [38]; (6) self-reported smoking cessation; (7) mood measured with the Standard Checklist 20-item Questionnaire (SCL-20) for depression [39] and the Standard Checklist 10-item Anxiety Measure (SCL-10A) [40]; and (8) a comprehensive measure of health status using the EuroQol 5-Dimension Questionnaire (EQ-5D) [41]. The SCL20 and SCL-10A are derived from a 90-question standard measure and have been used extensively to measure mood and anxiety as outcome measures in studies with people who have long-term conditions. The EQ-5D includes 5 questions asking about mobility, self-care, usual activities, pain and discomfort, and anxiety or depression. Each question has a 3-level response, and the responses are used to estimate a preference weight for that health status, presented as a single index value.

Details of number and duration of hospital admissions were measured by self-report and confirmed where possible by review of hospital discharge letters and central hospital admissions data. Records of deaths were obtained from general practices and further details were obtained, where necessary, from hospital records. Details of exacerbations of COPD were recorded on a record form held by all participants [28].

### Sample Size

The sample size calculations were based on the number of patients required to demonstrate a mean difference of 6.6 on the St George’s Respiratory Questionnaire between the two allocated groups, at 12-months from randomization (equivalent to 7.3 on SGRQ-C) [37]. We estimated the SD at 12.7 based on a study using the SGRQ-C [42]. For a power of 90% and significance level of .05 (2-sided), with 2:1 allocation between intervention and usual care and allowing for 10% loss to follow-up, we required 165 patients.

We also had 98% power to identify the difference in admissions to hospital at 3 months based on effect sizes of previous intensive interventions with this group of patients [7], and 52% power to detect the difference in admissions at 12 months based on a systematic review of interventions in COPD [43]. In both cases, a 5% loss to follow-up was assumed.

### Randomization

Participants were randomized with an allocation ratio of 2:1 intervention to usual care using Sortition V.1.2 [28]. The research nurse carried out randomization by accessing Sortition using a Web-browser on a tablet computer at the assessment visit only after completion of consent procedures and baseline measurements, including completion of the SGRQ-C.

### Trial Procedures

#### Recruitment

Potentially eligible patients were sent an invitation to participate in the trial. The invitation included a patient information booklet, a reply slip, and prepaid envelope. Patients who were interested in participating were asked to return their reply slips by post to the research team. The research nurse then contacted the patient by telephone to arrange an initial assessment visit. At this visit, eligibility was confirmed, written informed consent was obtained, and baseline data were collected for those consenting to participate.

All participants were assessed at baseline by a health care professional and had finished self-completed measures before randomization and intervention allocation. The use of medication by participants was recorded at the baseline and follow-up assessment visits. Information collected included type, dose, and frequency of COPD medication (tablets and inhalers) as well as a list of other medication taken. A detailed smoking history was taken at the baseline assessment visit; self-reported smoking status was recorded at subsequent assessments. All participants had either a written or tablet computer held action plan (uploaded as Multimedia Appendix 3) for use with changing symptoms, and were encouraged to ensure that, in line with standard practice, they had reserve supplies of antibiotics and steroids.

#### Patient Follow-Up and Retention

Patients remained in the trial for 12 months with assessments at a baseline visit, 3, 6, and 12 months. The primary outcome measure was collected at baseline, and 6 and 12 months after randomization. Secondary outcome measures were collected at baseline, 3, 6, and 12 months.

Postal reminders were sent before all follow-up assessment visit dates. The 3-month assessment was a telephone contact with patients. For patients allocated to standardized usual care, a reminder was posted before the assessment date. The 6 and 12 months visits were carried out either at home or at clinic.

Self-completed outcome measures were completed without guidance by the research team and before any further assessment or discussion of clinical care. Research and clinical teams were trained in the potential for measures to be biased by their interactions with participants. A record of all contacts with trial participants was kept to examine potential for interactions with patients not specified in the trial protocol.

All patients had the right to withdraw from the trial at any point, without providing a reason. Those patients who withdrew from receiving the intervention were asked if they would be willing to provide follow-up information within the trial at the 6- and 12-month assessment points. If patients declined, no further information was collected.

### Statistical Methods

The principal comparisons were performed on an intention-to-treat basis. The trial results are presented as comparative summary statistics (difference in response rates or means) with 95% CI. A linear mixed-effects model was used to analyze SGRQ-C including randomized group (intervention or control), time point (6 months and 12 months), baseline SGRQ-C score, age (analyzed continuously), gender, current smoking status (yes or no), severity of COPD (according to NICE clinical guidelines or GOLD classification, moderate or severe or very severe), and site as fixed effects and a patient-specific random intercept. Treatment-time interaction was included in the model to assess the treatment effect at 12 months. Higher scores reflect better quality of life compared with lower scores. Binary outcomes were analyzed using log-binomial regression, adjusting for covariates as described above. The threshold for statistical significance was less than *P*=.05 with no adjustments made for multiple testing among secondary outcomes.

The intervention effect was assessed by analysis of subgroups defined by severity of COPD, smoking status, hospital admission in the previous year, attending a pulmonary rehabilitation course in the previous year, and the presence or absence of live-in support. A full detailed statistical analysis plan was prepared before the final analysis by a trial statistician.

The EDGE COPD trial was carried out in conformance with the principles of the current version of the Declaration of Helsinki and the other regulations in force. The trial is registered at ISRCTN 40367841.

## Results

### Trial Progress and Baseline Characteristics of Participants

The CONSORT flow chart is presented in Figure 1. The reasons for patient withdrawal are also detailed in Figure 1. The first patient was randomized on June 26, 2013 and follow-up was completed on July 27, 2015.

Baseline characteristics of trial participants are summarized in Table 1 and in Multimedia Appendix 1. There were no relevant differences in characteristics between those assigned to EDGE and to usual care.

A total of 14 patients, who were allocated to receive the EDGE system, withdrew from the trial of whom 5 died: 7 withdrew from the usual care group with 4 deaths (Figure 1).

### Use of the System

Out of the 110 patients who were part of the intervention arm of the study, 100 patients were in the study for at least 180 days. Compliance with use of the system was a mean (SD) of 5.9 (1.1) days per week of use across all patients (range 1.4-7.0). Among the 100 patients, only 2 patients had a compliance of less than 3 times per week. The video clips offered to intervention group participants were used with varying frequency. The videos relating to mood and breathing exercises were reviewed more frequently than others (Multimedia Appendix 1). In total, 90% (99/110) of participants viewed at least one video. The mean (SD) number of videos viewed by participants was 5 (3.5) and the mean number of times a video was viewed was 22.5 (19.9). The mean (SD) number of accesses made by the nurse on the website to the data for each participant in the intervention group was 33.4 (SD 15.4, range 11-79), that is, an average of 2.78 accesses per patient per month.

### Primary Outcome

Quality of life as measured with the SGRQ-C improved in patients allocated to both the EDGE system and to usual care from baseline to 6 months, and again to 12 months. The estimated difference in SGRQ-C at 12 months (EDGE system−usual care) was −1.7 with a 95% CI of −6.6 to 3.2 (*P*=.49). SGRQ-C scores and changes over time are summarized in Table 2. Data were available on 84.5% (93/110) of patients in the EDGE system group, and from 85.7% (48/56) patients in the usual care group.

**Table 1:** Baseline characteristics of participants.

Characteristics		EDGE^a^ intervention n=110	Standard Care n=56
**Clinical data**				
	Male, n (%)	68 (61.8)	34 (60.7)
	Age, mean (SD^b^)	69.8 (9.1)	69.8 (10.6)
	BMI^c^, mean (SD)	28.6 (7.1)	29.1 (7.8)
	FEV1^d^, mean (SD)	47.4 (15.6)	50.1 (16.9)
	FEV1 or FVC^e^, mean (SD)	47.6 (11.3)	49.8 (11.5)
	Number of COPD^f^medications, median (IQR^g^)	5 (3-6)	5 (4-6)
	Number of other medications, median (IQR)	4 (2-7)	5 (2.5-8)
**Smoking history**				
	Current, n (%)	23 (20.9)	13 (23.2)
	Ex-smoker (<2 years), n (%)	17 (15.5)	8 (14.3)
	Ex-smoker (≥2 years), n (%)	70 (63.6)	35 (62.5)
**COPD severity**				
	Moderate, n (%)	41 (37.3)	23 (41.1)
	Severe or very severe, n (%)	69 (62.7)	33 (58.9)
**MRC^h^dyspnoea score, N (%)**				
	2	17 (15.5)	10 (17.9)
	3	74 (67.3)	39 (69.6)
	4	19 (17.3)	7 (12.5)
Comorbid conditions including high blood pressure, osteoporosis, high cholesterol, diabetes, heart disease, and depression), N (%)		89 (80.9)	47 (83.9)
**Patient reported outcome measures**				
	SGRQ-C (St George’s Respiratory questionnaire for COPD patients), mean (SD)	56.4 (19.7)	55.5 (16.2)
	SCL-10A^i^, median (IQR)	0.3 (0.1-0.9)	0.3 (0-0.5)
	SCL-20^j^, median (IQR)	0.53 (0.3-1.15)	0.68 (0.3-1.1)
	BMQ (Beliefs about Medicines Questionnaire), mean (SD)	24.6 (4.8)	25.3 (5.7)
	MARS (Medicines Adherence Report Scale), mean (SD)	23.4 (2.3)	22.5 (3.8)
	EQ-5D^k^Index, mean (SD)	0.62 (0.24)	0.63 (0.24)
	Deprivation score^l^, mean (SD)	22,440 (7951.9)	22,777 (7261.5)

^a^EDGE: sElf-management anD support proGrammE.

^b^SD: standard deviation.

^c^BMI: body mass index.

^d^FEV1: forced expiratory volume in 1 s.

^e^FVC: forced vital capacity.

^f^COPD: chronic obstructive pulmonary disease.

^g^IQR: interquartile range (25th, 75th percentiles).

^h^MRC: Medical Research Council.

^i^SCL-10A: Standard Checklist 10-item Anxiety Measure.

^j^SCL-20: Standard Checklist 20-item Questionnaire.

^k^EQ-5D: EuroQol 5-Dimension Questionnaire.

^l^On the basis of postcode with deprivation rankings accessed from the UK Office of National Statistics.

**Table 2:** Primary outcome—St George’s Respiratory Questionnaire for chronic obstructive pulmonary disease.

Primary Outcome		EDGE^a^intervention N=110	Standard care N=56
**SGRQ-C^b^**			
	Baseline, mean (SD^c^)	56.4 (19.7)	55.5 (16.2)
	6 months, mean (SD)	55.7 (20.2) N=98	54.3 (21.8) N=51
	12 months, mean (SD)	56.9 (19.5) N=93	56.8 (20.9) N=48
**Difference between groups^d^**			
	6 months, mean (95% CI; *P* value)	0.99 (−3.81 to 5.78; .69)	
	12 months, mean (95% CI; *P* value)	−1.74 (−6.65 to 3.16; .49)	

^a^EDGE: sElf-management anD support proGrammE.

^b^SGRQ-C: St George’s Respiratory Questionnaire for COPD.

^c^SD: standard deviation.

^d^From mixed effects model including randomized group (intervention or control), time point (6 months and 12 months), baseline SGRQ-C score, age (analyzed continuously), gender, current smoking status (yes or no), severity of COPD (according to NICE clinical guidelines or GOLD classification, moderate or severe or very severe), and site as fixed effects and a patient-specific random intercept. Higher scores reflect better quality of life compared with lower scores.

### Secondary Outcomes

Secondary outcomes are summarized in Table 3. Deaths did not differ between groups. Numbers of exacerbations did not differ overall between groups. The relative risk of hospital admission for EDGE was 0.83 (0.56-1.24, *P*=.37) compared with usual care. There was a significant difference in overall health status measured with the 5-Level EuroQol 5-Dimension Questionnaire (EQ-5D-5L) between groups 0.076 (0.009-0.14, *P*=.03), with better health status for the digital health group. There were fewer visits to the GP practice nurses 1.5 (digital health) versus 2.5 (usual care), *P*=.03 in comparison to the usual care group. The difference did not reach statistical difference for the median number of visits to general practitioners: 4.0 (digital health) versus 5.5 (usual care), *P*=.06. There was no difference in Beliefs about Medicines Questionnaire (BMQ) or self-reported medicines adherence (MARS, Medication Adherence Report Scale), and no differences in self-reported smoking cessation. Depression measured with the SCL-20 decreased in the EDGE group and increased in the standard care, but the difference in change between groups was not statistically significant. Anxiety measured with the SCL-10A was unchanged in the EDGE group, whereas increased in the usual care group, but again the difference in change between groups was not statistically significant.

**Table 3:** Secondary outcomes baseline to 12 months.

Secondary outcomes	Overall effect comparing EDGE^a^and usual care			
	EDGE intervention	Standard care	Adjusted treatment effect^b^(95% CI)	*P* value
Death rates, n (%)	6 (5.5)	4 (7.1)	1.7^c^(−11.0 to 4.9)	.73
Number with at least one admission, n (%)	38 (34.6)	23 (41.1)	0.83^d^(0.56-1.24)	.37
Number of exacerbations, median (IQR^e^)	1 (0-2)	1 (0-3)	1.21^f^(0.79-1.84)	.39
Change in BMQ^g^, mean (SD^h^)	−1.26 (5.33)	−0.80 (4.69)	−0.64^i^(−2.28 to 1.00)	.44
Medication Adherence Report Schedule, mean (SD)	0.17 (2.47)	0.33 (3.65)	0.10^j^(−0.57 to 0.77)	.77
Smoking cessation, n (%)	76 (81.7)	41 (85.4)	1.40^k^(0.24-7.96)	.71
Change in EQ-5D-5L^l^, mean (SD)	0.01 (0.21)	−0.08 (0.19)	0.076^i^(0.009-0.14)	.03
Change in SCL-20^m^, mean (SD)	−0.04 (0.46)	0.14 (0.56)	−0.13^i^(−0.29 to 0.03)	.10
Change in SCL-10A^n^, mean (SD)	0.03 (0.59)	0.13 (0.43)	0.002^i^(−0.19 to 0.19)	.98
Change in lung function, mean (SD)	−0.78 (10.23)	−1.40 (5.67)	0.32^i^(−5.58 to 6.22)	.91
Number of GP^o^contacts (surgery), median (IQR)	4 (2-7)	5.5 (2-10)	-	.06
Number of nurse contacts (surgery), median (IQR)	1.5 (1-3)	2.5 (1-7)	-	.03

^a^EDGE: sElf-management anD support proGrammE.

^b^Adjusted for baseline values and minimization factors.

^c^Difference in proportion.

^d^Relative risk.

^e^IQR: interquartile range.

^f^Incidence rate ratio.

^g^BMQ: Beliefs about Medicines Questionnaire.

^h^SD: standard deviation.

^i^Difference in change in mean.

^j^Difference in mean.

^k^Odds ratio.

^l^EQ-5D-5L: 5-Level EuroQol 5-Dimension Questionnaire.

^m^SCL-20: Standard Checklist 20-item Questionnaire.

^n^SCL-10A: Standard Checklist 10-item Anxiety Measure.

^o^GP: general practitioner.

**Figure 2:**
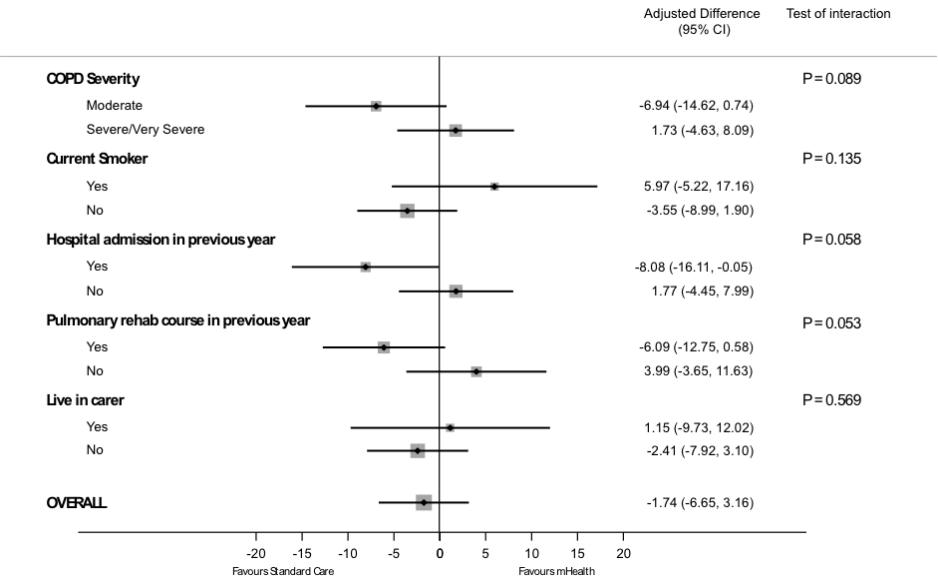
Subgroup analyses for primary outcome.

### Subgroup Analysis of Primary Outcome

Prespecified subgroup analyses for the change in SGRQ-C between intervention groups are summarized in Figure 2. Severity of COPD, current smoker, and no previous hospital admissions in the last year appeared to favor the EDGE system, although interaction terms did not reach statistical significance.

### Safety Data

There was no difference in the rates of adverse events or serious adverse events between groups, and none of these events was deemed to be trial or intervention-related. Primary care clinicians were contacted on the same day that a clinically important safety alert was identified.

### Costs

The cost of each tablet computer used was £319 and the cost of the pulse oximeter probe was £399. Reviewing patient data on the clinician website using the prioritization algorithms took a median (IQR, interquartile range) of 1.2 h per session (2.9), with a median number of participants accessed for each session of 16 (54). The cost of a hospital admission was estimated as £2900, a GP appointment as £36, and a practice nurse appointment as £11 [44]. The respiratory clinicians reviewing the data reported that up to one phone call a week to a general practitioner, practice or respiratory nurse was required, following patient data review.

## Discussion

### Principal Findings

This trial provides evidence that use of a tablet computer-based system of monitoring and self-management support does not impact, either positively or negatively on COPD-specific health status over a period of 12 months. However, the finding of a significant improvement in prespecified secondary outcomes supports a beneficial impact on broader measures of health status and number of visits to practice nurses. Although the trial did not test the intervention in a sufficiently large sample to detect a significant difference between patients allocated to intervention and usual care, the effect sizes observed for an improved depression score, reductions in hospital admissions, and reduction in visits to a general practitioner compared with usual care suggest a need for further evaluation.

This trial was carried out in a representative population of individuals with moderate to very severe COPD using minimal exclusion criteria. It employed state-of-the art trial management including use of minimization to balance groups, an unbalanced allocation to allow the possibility of examining the effect of the EDGE platform across a wide range of participants, and measures to avoid contamination. In contrast to several recently reported trials of telehealth in COPD, the equipment was affordable and did not require specialist installation. Features of the EDGE platform addressing previous concerns about telehealth systems include the use of a generic tablet computer, access through icons rather than a keyboard, use of Bluetooth sensors with error-checking algorithms to ensure high-quality data, support for patient interpretation of data, and tailored self-management content [45]. The study protocol mandated patient contact when patient-specific, rather than study-wide thresholds were reached. The prioritization algorithms used to implement personalized alerting ensured that the workload of the nurses reviewing the patient data was kept to manageable levels (a median of 1.2 h per session, with a median of 16 patients accessed per session; 2 sessions per week). This trial addresses previous concerns about the lack of well-designed randomized controlled trials with appropriate follow-up raised in a recent review of telemedicine interventions for COPD [46].

The use of a self-reported primary outcome measure is a potential limitation, but this was completed before measurement and other data collection at the final visit. Other limitations of the trial design that could be addressed in future work include moving to the use of the updated GOLD classification to characterize participants at baseline. Although no adjustment for testing of multiple secondary outcomes was made, all were prespecified.

As with other trials [22,47,48], and in line with a systematic review of telehealth trials [49], an impact on COPD-specific health status was not observed. Although COPD-specific health status is an important aspect of COPD that has been shown to be responsive to group and one-to-one interventions [50], a digital health intervention used on its own may not replace a more intensive and personalized intervention for many patients. However, other outcomes are also important, including broader measures of health status, psychological health measures, and use of hospital services. It is possible that use of the intervention in clinical practice may lead to changes in the behavior of doctors and nurses, and the way that the health system responds to patients. These changes could further improve outcomes. Further studies to evaluate the system may therefore need to be carried out with clustering of intervention delivery by functional units, for example, individual primary care sites.

The EDGE system was used daily, to send pulse oximeter and diary data, by over 80% of the trial patients. The use of algorithms on the tablet computer to assess quality of monitoring data minimized false alerts from the system. The limited impact observed on some secondary outcomes in this trial could be mediated by regular self-monitoring and review of charted data together with use of the educational and motivational material available on the tablet computer, and, perhaps for some, the wider use of the tablet computer to communicate with family and friends. The effects observed in this trial could be further enhanced by use of better predictive algorithms guiding self- and clinician-management. Monitoring additional parameters (eg, mobility) and prompting additional measurement may also improve algorithm performance. The comprehensive data from this trial, where the external monitoring of data was restricted to ensuring patients’ safety, provide a source for predictive modeling [24]. Predictive modeling from these data, along with additional parameters (including mobility) will be integrated into strategies for early treatment of exacerbations [51]. Strategies will include prompting for additional measurement to improve the accuracy of predictions, graded alerts to patients prompting clinical review or starting treatment, and alerts to clinicians to proactively contact patients.

The trial intervention uses a novel implementation of telehealth using a nonproprietary tablet computer designed for integration into day-to-day life and clinical care. In addition to being nonobtrusive, it provides, at relatively low cost in relation to previous telehealth systems, facilities for monitoring, communication, self-management support, and education delivery. The development of the system was carried out iteratively using best practice to involve patients, engineers, and clinicians in repeated testing and assessment [52]. Future iterations of the technology could extend beyond the limited implementation of the EDGE system used in this study [36], for example, in integration with electronic health records.

The underlying approach to implementing digital health within this trial was to provide a system focused around the needs of the patient, with collection of data that can be analyzed over a period of time and used to inform future management. Data from this trial will be used to evaluate the potential of patient-specific tailored alerts, as well as informing the design of multicenter trials to explore cost-effectiveness and potential for reduction in hospital admissions [46]. The dataset would also be available to test algorithms derived from other projects.

### Conclusions

Although this clinical trial does not provide evidence for an effect on COPD-specific health status from the EDGE digital health system in comparison with usual care, there may be an overall benefit to patients through better overall health status. If an intervention with the effect sizes for reduced hospital admissions and primary care visits of the magnitude reported in this trial were implemented at scale, it would make an important contribution to monitoring and self-management support for people with moderate to very severe COPD.

## Multimedia Appendix 1

Supplementary tables and figures.

## Multimedia Appendix 2

TIDIER Checklist (Intervention description).

## Multimedia Appendix 3

Action plan provided to all trial participants to manage disease exacerbations.

## Multimedia Appendix 4

CONSORT E-Health Checklist V 1.6.1.

## Abbreviations

BMQ: Beliefs about Medicines Questionnaire
COPD: chronic obstructive pulmonary disease
EDGE: sElf-management anD support proGrammE
EQ-5D-5L: 5-Level EuroQol 5-Dimension Questionnaire
IQR: interquartile range
MARS: Medication Adherence Report Scale
