# Supplementary material for: Self-Management Support Using a Digital Health System Compared With Usual Care for Chronic Obstructive Pulmonary Disease: Randomized Controlled Trial
Source: J Med Internet Res. 2017 May 3;19(5):e144. doi: 10.2196/jmir.7116 (PMC5438446; doi:10.2196/jmir.7116)
Supplement: Supplementary file 1 [file jmir_v19i5e144_app1.pdf]

Supplementary tables

Supplementary Material: List of brief video clips made available as part of the self-management support component of the EDGE-System

"The titles of the included videos were:

1 How to use the Handihaler";
2 "How to use the Accuhaler";
3 "How to use the Metered Dose Inhaler";
4 "How to use the Turbuhaler";
5 "Pulmonary rehabilitation importance" [a];
6 "Stay active";
7 "How to use the Aerochamber";
8 "Manage my mood";
9 "Manage my breathing";
10 "Manage my worry"
11 "How to use the Respimat"
12 "Relaxation technique";
13 "My breathing exercises"; and
14 "How to use the Volumatic".

[a]The pulmonary rehabilitation video was developed by Oxford Health NHS Foundation Trust to supplement the evidence-based course which many of the trial participants had already attended. Other videos were based on standard clinical practice with the aim of supplementing advice already received."

Supplementary Table 1: Definition of an exacerbation

The number of recorded exacerbations defined as episodes in which antibiotics or oral steroids were prescribed or in which the patients were seen in the accident and emergency department or admitted to hospital in the presence of an acute change in respiratory symptoms (defined as the presence of at least two symptoms, one of which should be major (major symptoms: change in sputum; more breathless; chest tight. minor symptom: unwell, tired, temperature, a cold) or a report of a patient taking more salbutamol, either blue inhaler or by nebuliser, for at least 48 hours).

Major symptoms	Change in sputum;<br>More breathless;<br>Chest tight.
Minor symptom	Unwell,<br>Tired,<br>Temperature,<br>A cold (URTI)

Supplementary Table 2: Detailed baseline characteristics of participants

			mHealth N=110	Standard Care N=56
**Clinical data**				
Male		N (%)	68 (61.8)	34 (60.7)
Age		Mean (SD)	69.8 (9.1)	69.8 (10.6)
BMI		Mean (SD)	28.6 (7.1)	9.1 (7.8)
FEV1		Mean (SD)	47.4 (15.6)	50.1 (16.9)
FEV1/FVC		Mean (SD)	47.6 (11.3)	49.8 (11.5)
Number of COPD medications		Median (IQR)	5 (3, 6)	5 (4, 6)
Number of other medications		Median (IQR)	4 (2, 7)	5 (2.5, 8)
Smoking history				
	Current	N (%)	23 (20.9)	13 (23.2)
	Ex-smoker (< 2 years)	N (%)	17 (15.5)	8 (14.3)
	Ex-smoker (≥ 2 years)	N (%)	70 (63.6)	35 (62.5)
COPD Severity				
	Moderate	N (%)	41 (37.3)	23 (41.1)
	Severe/Very severe	N (%)	69 (62.7)	33 (58.9)
MRC Dyspnoea score				
	2	N (%)	17 (15.5)	10 (17.9)
	3	N (%)	74 (67.3)	39 (69.6)
	4	N (%)	19 (17.3)	7 (12.5)
Co-morbid conditions including high blood pressure, osteoporosis, high cholesterol, diabetes, heart disease and depression)		N (%)	89 (80.9)	47 (83.9)
**Patient reported outcome measures**				
SGRQ-C (St George's Respiratory questionnaire for COPD patients)		Mean (SD)	56.4 (19.7)	55.5 (16.2)
SCL-10 A		Median (IQR)	0.3 (0.1, 0.9)	0.3 (0, 0.5)
SCL-20		Median (IQR)	0.53 (0.3, 1.15)	0.68 (0.3, 1.1)
BMQ (Beliefs about Medicines Questionnaire)		Mean (SD)	24.6 (4.8)	25.3 (5.7)
MARS (Medicines Adherence Report Scale)		Mean (SD)	23.4 (2.3)	22.5 (3.8)
EQ-5D Index		Mean (SD)	0.62 (0.24)	0.63 (0.24)
Deprivation score*		Mean (SD)	22440 (7951.9)	22777 (7261.5)
**Use of computers and mobile phones**				
Participant has computer		N (%)	97 (88.2)	54 (96.4)
Frequency of use of computer				
	Never	N (%)	11 (10.0)	5 (8.9)
	Hardly ever	N (%)	3 (2.7)	4 (7.1)
	Sometimes	N (%)	2 (1.8)	5 (8.9)
	Quite frequently	N (%)	16 (14.6)	6 (10.7)
	Everyday	N (%)	54 (49.1)	24 (42.9)
	N/A	N (%)	13 (11.8)	2 (3.6)
	Missing	N (%)	11 (10.0)	10 (17.9)

Participant has a mobile phone	N (%)	97 (88.2)	54 (96.4)

			mHealth N=110	Standard Care N=56
Frequency of use of mobile phone				
	Never	N (%)	4 (3.6)	3 (5.4)
	Hardly ever	N (%)	23 (20.9)	14 (25.0)
	Sometimes	N (%)	17 (15.5)	9 (16.1)
	Quite frequently	N (%)	15 (13.6)	5 (8.9)
	Everyday	N (%)	38 (34.6)	23 (41.1)
	N/A	N (%)	13 (11.8)	2 (3.6)
Mobile phone used for?				
	Only calls/texting	N (%)	79 (71.8)	45 (80.4)
	Calls, texting and internet	N (%)	17 (15.5)	7 (12.5)
	N/A	N (%)	13 (11.8)	2 (3.6)
	Missing	N (%)	1 (0.9)	2 (3.6)
**Pulmonary rehabilitation**				
Previously attended a pulmonary rehabilitation course		N (%)	74 (67.3)	27 (48.2)
If yes, how long ago?				
	<6 months ago	N (%)	34 (30.9)	12 (21.4)
	6-12 months ago	N (%)	14 (12.7)	2 (3.4)
	>12 months ago	N (%)	26 (23.6)	13 (23.2)
	N/A	N (%)	36 (32.7)	29 (51.8)
Referred for pulmonary rehabilitation		N (%)	19 (17.3)	11 (19.6)
Have a carer		N (%)	35 (31.8)	18 (32.1)
If yes do they live with participant?				
	Yes	N (%)	26 (23.6)	12 (21.4)
	No	N (%)	9 (8.2)	6 (10.7)
	N/A	N (%)	75 (68.2)	38 (67.9)
**Use of community health and hospital servicers**				
Seen by community respiratory nurse team		N (%)	41 (37.3)	21 (37.5)
Total number of hospital admissions in past 12 months				
	0	N (%)	58 (52.7)	38 (67.9)
	1	N (%)	33 (30.0)	14 (25.0)
	2	N (%)	14 (12.7)	2 (3.6)
	3	N (%)	3 (2.7)	1 (1.8)
	4	N (%)	1 (0.9)	1 (1.8)
	5	N (%)	0	0
	6	N (%)	1 (0.9)	0
At least one hospital admission in last 12 months		N (%)	52 (47.3)	18 (32.1)
Number of times seen GP in surgery in last 3months				
	Median (IQR)		1.5 (0, 3)	2 (1, 3)
	0	N (%)	32 (29.1)	12 (21.4)
	1	N (%)	23 (20.9)	11 (19.6)
	2	N (%)	23 (20.9)	13 (23.2)
	≥3	N (%)	32 (29.1)	20 (35.7)
Number of times seen GP at home in last 3 months				
	Median (IQR)		0 (0, 0)	0 (0, 0)
	0	N (%)	99 (90.0)	49 (87.5)
	1	N (%)	7 (6.4)	4 (7.1)
	≥2	N (%)	4 (3.6)	3 (5.4)
Number of unplanned GP contacts in last 3 months				
	Median (IQR)		0 (0, 1)	0 (0, 1)
	0	N (%)	74 (67.3)	41 (73.2)
	1	N (%)	22 (20.0)	5 (8.9)

			mHealth N=110	Standard Care N=56
	≥2	N (%)	14 (12.7)	10 (17.9)
Number of times seen by nurse in surgery in last 3 months				
	Median (IQR)		1 (0, 1)	1 (0, 2)
	0	N (%)	54 (49.1)	27 (48.2)
	1	N (%)	37 (33.6)	13 (23.2)
	≥2	N (%)	19 (17.3)	16 (28.6)
Number of times seen by nurse at home in last 3 months				
	0	N (%)	108 (98.2)	55 (98.2)
	≥1	N (%)	2 (1.8)	1 (1.8)
Number of unplanned nurse contacts in last 3 months				
	0		106 (96.4)	53 (94.6)
	1	N (%)	4 (3.6)	3 (5.4)
		N (%)		
Number of times seen by community respiratory nurse team in last 3 months				
	Median (IQR)		88 (80.0)	47 (83.9)
	0	N (%)	22 (20.0)	9 (16.1)
	≥1	N (%)		
Number of times seen at A&E in last 3 months				
	Median (IQR)		91 (82.7)	52 (92.9)
	0	N (%)	19 (17.3)	4 (7.1)
	≥1	N (%)		
Number of admissions to inpatient care in last 3 months				
	0	N (%)	93 (84.6)	50 (89.3)
	≥1	N (%)	17 (15.4)	6 (10.7)
Days spent in hospital in last 3 months				
	0	N (%)	91 (82.7)	50 (89.3)
	1-3	N (%)	8 (7.3)	3 (5.4)
	≥4	N (%)	11 (10.0)	3 (5.4)
Number of times attended respiratory outpatient clinic in last 3 months				
	0	N (%)	67 (60.9)	38 (67.9)
	≥1	N (%)	43 (39.1)	18 (32.1)
Number of times attended pulmonary rehabilitation class in last 3 months				
	Median (IQR)		0 (0, 3)	0 (0, 0)
	0	N (%)	78 (70.9)	45 (80.4)
	1-11	N (%)	19 (17.3)	6 (10.7)
	≥12	N (%)	13 (11.8)	5 (8.9)
Number of times received physiotherapy in last 3 months				
	0	N (%)	96 (87.3)	54 (96.4)
	≥1	N (%)	14 (12.7)	2 (3.6)
Number of times received occupational therapy in last 3 months				
	0	N (%)	105 (95.4)	55 (98.2)
	≥1	N (%)	5 (4.6)	1 (1.8)

* Based on postcode with deprivation rankings accessed from http://www.streetgames.org/www/sgplus/content/areas-deprivation-postcode for individual searches (inputting postcode links to ONS website). Bulk lists (IMD 2010) were accessed via http://dclgapps.communities.gov.uk/imd/imd-by-postcode.html
IQR = Inter quartile range (25th, 75th Percentiles)

Supplementary Figure

Probably density curve for clinician time spent each session reviewing data

Vertical (y) axis – probability of given session length

Horizontal (x) axis - time for each session

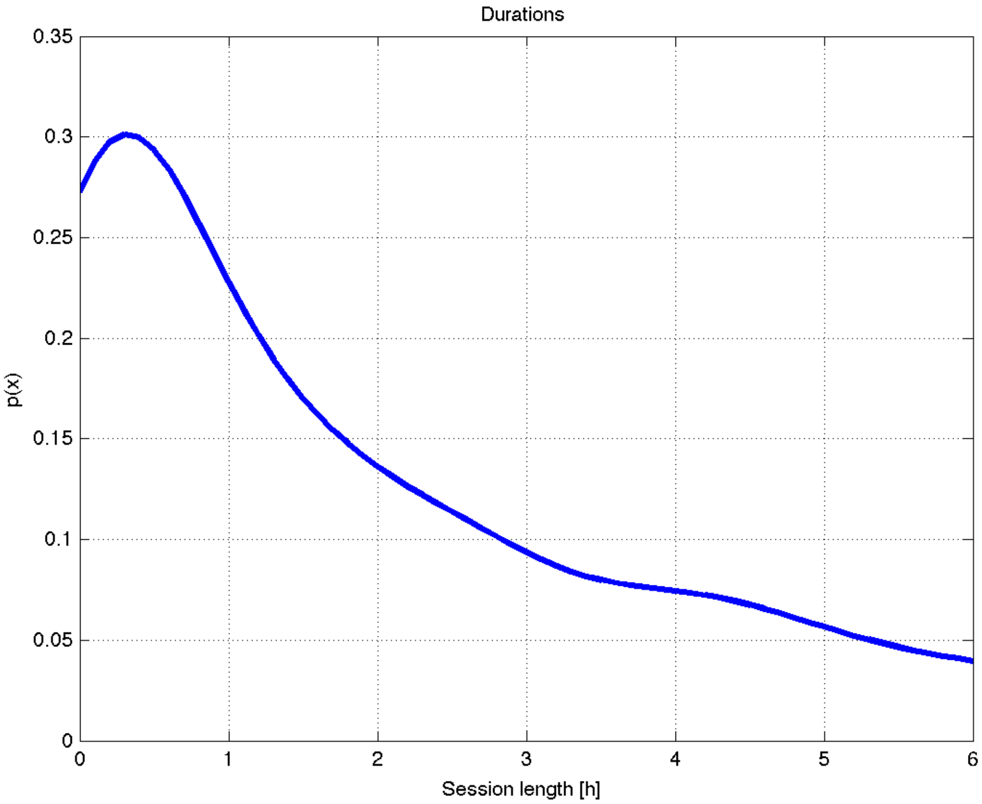

Footnote. Duration of sessions was estimated from time for which the system was active. This does not account for time spent when logged on but not using the system – so estimates are conservative. Therefore, for at least 60% of the sessions, the nurse was logged on for one hour or less.
