# Supplementary material for: Self-Management Support Using a Digital Health System Compared With Usual Care for Chronic Obstructive Pulmonary Disease: Randomized Controlled Trial
Source: J Med Internet Res. 2017 May 3;19(5):e144. doi: 10.2196/jmir.7116 (PMC5438446; doi:10.2196/jmir.7116)
Supplement: Supplementary file 2 [file jmir_v19i5e144_app2.pdf]

Supplementary Table: Checklist for development of the EDGE Digital Health Intervention

**1.**	**Brief name**	Usual care	EDGE
**2.**	**Rationale or theory**	We developed and tested the EDGE intervention to support patients with COPD in monitoring their health and to provide information and education about their condition. Tablet computers bring together communications and computing capability. We developed a digital platform with which to collect data from patients and deliver multi-media support to patients and integrated this with clinical care to deliver the EDGE intervention.[1] The intervention draws on ideas about how people think about their illness to guide the way symptoms and clinical measurements are gathered from patients with moderate to severe COPD. This data is analysed and displayed to patients alongside educational videos and treatment plans.[2] The aim of communicating this information is to support patient self-management by enabling a better understanding of the course of disease around exacerbations.[3] The data also allows remote monitoring for safety by nurses. Key principles underlying the development of the platform for intervention delivery included ease of use for people less experienced with computers (large icons and no keyboard needed for data entry) and ensuring reliable data (video instructions to encourage correct technique in using the equipment and repeat measurement where needed to assure data quality).[3]	
**3.**	**Materials**	Printed information pack including:<br>• how to use medications<br>• when medications should be used,<br>• a self-management plan with written guidelines on what to do and whom to contact if experiencing an exacerbation and<br>• dietary advice<br>• invitation to attend a pulmonary rehabilitation course	• Brief information booklet giving details of the use of the EDGE application<br>• Tablet computer running the EDGE application<br>• Bluetooth enabled pulse oximeter linked to tablet computer.
**4.**	**Procedures**	• Written record of use of antibiotics and contacts with GP, nurse and hospital	• Daily measurement of pulse, oxygen saturation and self-reported symptoms<br>• Review of personalised plans for self-

			management and treating an exacerbation of their condition;<br>• Review of brief video clips and text-based material providing additional information about COPD and treatments (including medicines use and inhaler technique), with facility for reviewing targeted videos addressing these areas;<br>• Review of educational advice on managing COPD, smoking cessation, diet, physical activity;<br>• Monthly screening for depression and anxiety;<br>• The facility to receive a brief message from a respiratory nurse (e.g. encouragement to seek influenza vaccination or advising a change of batteries for the pulse oximeter)<br>• Written record of use of antibiotics and contacts with GP, nurse and hospital
**5.**	**Intervention provider**	Usual clinical care received from GP, practice nurse and community nursing service.	Android tablet computer (Samsung Galaxy Tab) running the application software and Bluetooth-enabled oximeter probe (Nonin, PureSAT, 956OBT, Nonin Medical Inc, Plymouth, Minnesota, USA). The server is hosted behind NHS firewalls by an NHS Hospital Trust.[3]
**6.**	**Modes of delivery**	Not applicable	Participants were briefly instructed on the use of the EDGE platform by the research nurse and given a brief information booklet detailing its use. Patients were informed that the EDGE platform was not a replacement for their usual clinical care, and that in the event of deterioration in their health they should contact their general practitioner or community respiratory nurse as usual. Participants continued to input their symptom data and clinical recordings

			daily throughout the duration of the trial. Patients were informed that the EDGE system was not a replacement for their usual clinical care, and that in the event of deterioration in their health they should contact their general practitioner or community respiratory nurse as usual. [4]
**7.**	**Location where intervention occurred**	Out of hospital (home)	Wherever ever participant and their tablet computer and Nonin pulse oximter were located (real world)
**8.**	**Number of times intervention was delivered over what time period**	• Phone call at three months<br>• Visit from nurse at six months	• Daily use of EDGE platform to record symptoms and oxygen saturation and pulse.<br>• Phone call at three months<br>• Visit from nurse at six months
**9.**	**What, why, when, how intervention was personalised or adapted**	Usual clinical care received from GP, practice nurse and community nursing service.	Care plan and list of medications, and associated leaflets were individualised. The appropriate videos and leaflets were uploaded by wireless.<br>An elevated score on the PHQ and GAD screening questions led to a prompt to view videos dealing with emotional well being. Every 4 weeks, beginning 2 weeks after initial use, the platform presents a four-item screening measure, the Patient Health Questionnaire two-item measure (PHQ-2) and the General Anxiety Disorder two-item measure (GAD-2).[5] If either the depression or anxiety score is >1 then the relevant full questionnaire, PHQ-8[6] or GAD-7,[7] is presented for completion.
**10.**	**Modifications during the trial**	Nil	Nil

**11.**	**Planned intervention delivery**	Not applicable	Daily uploading of data<br>All videos used
**12.**	**Actual intervention delivery**	Not applicable	Compliance with use of the system was 5.9 days a week of use across all patients (5.9 ± 1.1 range: 1.4-7.0). Among the 100 patients, only 2 patients had a compliance of less than 3 times/week. Nineteen videos out of 21 were viewed by trial participants in the EDGE intervention group a total of 579 times (min:1, max:123).

References

[1] A. Farmer, C. Toms, M. Hardinge, V. Williams, H. Rutter, and L. Tarassenko, “Self-management support using an Internet-linked tablet computer (the EDGE platform)-based intervention in chronic obstructive pulmonary disease: protocol for the EDGE-COPD randomised controlled trial.,” *BMJ Open*, vol. 4, no. 1, pp. e004437–e004437, 2014.

[2] V. Williams, J. Price, M. Hardinge, L. Tarassenko, and A. Farmer, “Using a mobile health application to support self-management in COPD: a qualitative study.,” *Br.J.Gen.Pract.*, vol. 64, no. 624, pp. e392–400, Jul. 2014.

[3] M. Hardinge, H. Rutter, C. Velardo, S. A. Shah, V. Williams, L. Tarassenko, and A. Farmer, “Using a mobile health application to support self-management in chronic obstructive pulmonary disease: a six-month cohort study,” *BMC Med.Inform.Decis.Mak.*, pp. 1–10, Jun. 2015.

[4] S. A. Shah, C. Velardo, O. J. Gibson, H. Rutter, A. Farmer, and L. Tarassenko, “Personalized alerts for patients with COPD using pulse oximetry and symptom scores.,” *Conf Proc IEEE Eng Med Biol Soc*, vol. 2014, pp. 3164–3167, 2014.

[5] B. Löwe, I. Wahl, M. Rose, C. Spitzer, H. Glaesmer, K. Wingenfeld, A. Schneider, and E. Brähler, “A 4-item measure of depression and anxiety: Validation and standardization of the Patient Health Questionnaire-4 (PHQ-4) in the general population,” *Journal of Affective Disorders*, vol. 122, no. 1, pp. 86–95, Apr. 2010.

[6] K. Kroenke, T. W. Strine, R. L. Spitzer, J. B. W. Williams, J. T. Berry, and A. H. Mokdad, “The PHQ-8 as a measure of current depression in the general population,” *Journal of Affective Disorders*, vol. 114, no. 1, pp. 163–173, Apr. 2009.

[7] R. L. Spitzer, K. Kroenke, J. B. W. Williams, and B. Löwe, “A Brief Measure for Assessing Generalized Anxiety Disorder: The GAD-7,” *Arch.Intern.Med.*, vol. 166, no. 10, pp. 1092–1097, May 2006.
