# Supplementary material for: Self-Management Support Using a Digital Health System Compared With Usual Care for Chronic Obstructive Pulmonary Disease: Randomized Controlled Trial
Source: J Med Internet Res. 2017 May 3;19(5):e144. doi: 10.2196/jmir.7116 (PMC5438446; doi:10.2196/jmir.7116)
Supplement: Supplementary file 3 [file jmir_v19i5e144_app3.pdf]

Oxford Health NHS
NHS Foundation Trust

Staying Healthy with CO[illegible]
(Chronic Obstructive Pulmonary Disease)

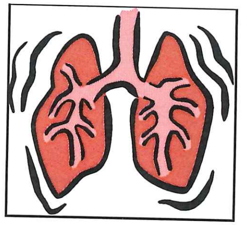

Handy hints and tips to enable you to stay as healthy as possible
Please read and contact our service if you have any questions or concerns

Keeping fit and healthy

- Make exercise a regular activity – try to walk every day if possible
- Take all of your prescribed medication regularly – speak to your nurse or doctor if you are unsure what to take
- Have a flu vaccine every year (you should also have a pneumonia vaccine once in your life only)
- Eat well – try to eat a balanced diet with plenty of fruit and vegetables. If eating makes you breathless try to eat smaller meals, sit at a table to eat and use your oxygen at meal times if you are prescribed it
- Speak to your GP if you are losing weight unintention-ally
- Drink at least 4 pints of fluid per day (unless you have been told to restrict fluids)
- Try to get a good nights sleep

Changes in the weather

- Hot/dry days – use fans, drink plenty, keep cool, use blue inhaler when breathless, avoid areas with lots of dust/fumes (bus stations etc)
- Cold/damp days – avoid being outdoors when misty and damp. Keep warm – hats, warm coats, gloves and scarves when outdoors. Keep the house warm

Exercising to stay healthy

People with breathing difficulties sometimes avoid exercise as they worry about becoming breathless. But it is not actually harmful as long as the exercise is introduced gradually and sensibly.

Regular exercise can improve the condition of your lungs and can greatly benefit your wellbeing and enjoyment of life.

Be on the lookout for changes and seek help if you become more unwell and please read Managing a 'Flare-up' Leaflet

Be careful what you breathe

- Stop smoking and avoid smoky places
- Try to avoid places with lots of exhaust fumes
- Avoid dust – damp dust and open windows when dusting/vacuuming
- Open windows if using strong smelling cleaners and avoid plug-in or spray air fresheners or candles
- Wear a mask – if going to do something that creates a lot of dust e.g. cleaning out pets' cages, decorating, sanding wood, etc.
- Try to avoid people with coughs and colds

Breathing exercises

There are various breathing exercises you can do to help. Please speak to your Respiratory Nurse so they can advise you on the best exercises for you

Ask your GP about
Pulmonary Rehabilitation.
stock at home.

When to seek help:-

- If you are deteriorating/symptoms are severe
- If you cough up any blood
- If you notice new or worsening
- ankle swelling especially if it doesn't go down overnight
- If you get any new chest pain
- If you cannot cope with this episode alone

If you need to go to hospital please try to remember to take all of your medication with you.

Oxford Health NHS
NHS Foundation Trust

Managing Flare-Ups of COPD (Chronic Obstructive Pulmonary Disease)

Please read and contact our service if you have any questions or concerns

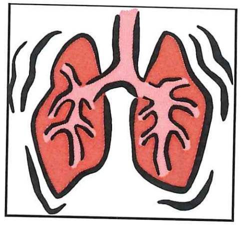

What is a 'flare-up'?

Many people with COPD have times when their usual symptoms become worse, for example finding it harder to breathe. This is called an exacerbation or 'flare-up'. This can often happen when the weather suddenly gets colder.

Recognising a 'flare-up'.

These are the symptoms that you might experience:-

- Worsening shortness of breath
- Worsening cough
- Increased sputum/phlegm
- Changes in the colour of sputum/phlegm
- Increased tiredness
- Developing a temperature and fever
- Developing a cold or viral infection
- Decrease in your usual activity
- More breathless than normal doing usual activities

Starting your treatment as Quickly as possible may help you to recover faster

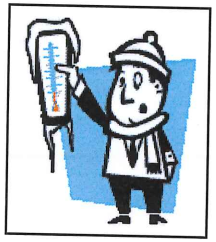

Take Steps 1 and 2 Together

Step 1 - Increase your reliever Inhaler (BLUE)

This will relieve breathlessness.
Always use a spacer if you have one. It may be that you will need to use this inhaler every 2-3 hours.

Increase your Blue Inhaler
to ______________________
__________times a day

Step 2 - If breathlessness is not relieved by Step 1, then:-

Start Prednisolone (Steroid) tablets

Take **Six x 5mg tablets** all together. It does not matter at what time of the day.

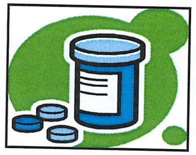

Then complete the course by taking **six** tablets in the morning for the following **six** days.

Step 3 - Starting Antibiotics

Only take if you have changes in the colour of your sputum (phlegm)

These may be Amoxicillin or another antibiotic prescribed by your GP.

Follow the instructions on your tablets and make sure you complete the course.

Step 4 - Inform your GP

Inform your GP when you start the stand-by medication.

If after 2 days better contact 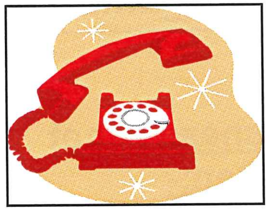 **you do not feel any your GP.**

Arrange a repeat prescription for the stand-by medication used. You will always need to keep a

When to seek help:-

- If you are deteriorating/symptoms are severe
- If you cough up any blood
- If you notice new or worsening
- ankle swelling especially if it doesn't go down over-night
- If you get any new chest pain
- If you cannot cope with this episode alone

If you need to go to hospital please try to remember to take all of your medication with you.

Oxford Health NHS
NHS Foundation Trust

Inhaler	Breakfast	Lunch	Dinner	Bedtime

Oxford Health NHS
NHS Foundation Trust

HOW TO USE THE METERED DOSE INHALER

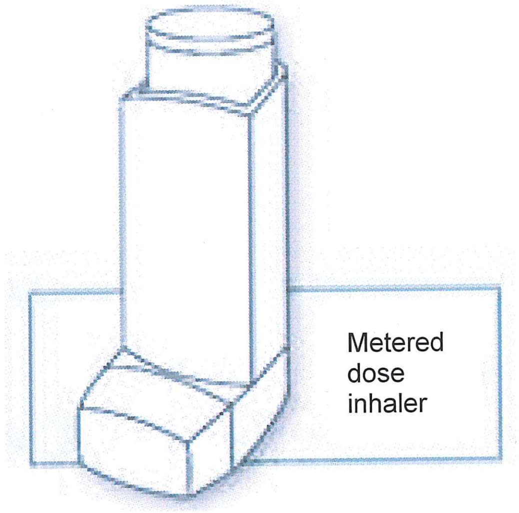

1. Shake the inhaler.
2. Hold the inhaler upright and remove the cap.
3. Breathe out gently.
4. Put mouthpiece in mouth and at start of inspiration, which should be slow and deep, press canister down and continue to inhale deeply.
5. Hold breath for 10 seconds, or as long as possible then breathe out slowly.
6. Wait for about 30 seconds before repeating steps 1-5.

Oxford Health NHS
NHS Foundation Trust

ABOUT YOUR HANDIHALER

It is important you use the inhaler correctly to make sure that you have the full dose of the medication you need.

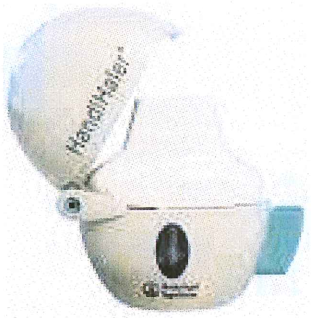

How Does is Work?
The Handihaler is used to provide Tiotropium (Spiriva) and the dose is one capsule per day as the effects are sustained for 24 hours. It is a drug to relax muscles of the airways.

How do I use it?

1. Open cover
2. lift up white mouth piece
3. Insert 1 green/blue capsule
4. Push mouthpiece back down until a click is heard
5. Press green button in once to pierce capsule
6. Take a slow breath out
7. Put mouthpiece in your mouth and breathe in quickly with mouth around inhaler mouthpiece and hold for 10 seconds (or as long as is comfortable).
8. Repeat a breath in with the same capsule if necessary to clear all of the powder, check the capsule, if powder remains seek advice on your technique.

Date: July 2009. Review Date: July 11
Author: Respiratory Team Birmingham University Hospital

Eating Well for Your Lungs

You have been given this leaflet to help you eat well and keep as healthy as possible.

Your diet and nutritional intake are very important when you have a disease like COPD.

COPD can have an effect on your whole body. It is therefore important to eat a balanced and varied diet to help maintain your strength and fitness, as well as help your body fight infections.

When you are feeling well and strong, healthy eating can keep you feeling this way.

It is important to think about your weight. If you are very overweight your heart and lungs have to work harder to supply oxygen to your body. Likewise if you are too thin you are more at risk from infections.

Ask your nurse what your Body Mass Index (BMI) is. If it is between 20 and 30, that is a healthy weight for someone with COPD.

If you find you are struggling to eat well and find that you are losing weight it is a good idea to mention this to your doctor or nurse.

Protein Foods

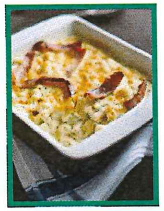

For healthy strong muscles (including those that help with breathing)

- Try to take a portion of a protein food with at least 2 meals during the day, e.g. lunch and dinner
- Foods that provide us with protein include: meat, fish, eggs, dairy products (cheese, yogurt, milk), tofu, beans, and lentils

Carbohydrate Foods

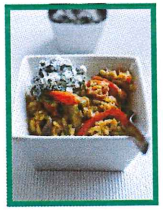

For energy

- Starchy foods include: potatoes, bread, pasta, rice, cereals - always include a starchy food at each meal
- Sugary foods include: cakes, biscuits, sweets, fizzy drinks - try to take in the diet occasionally.

Fruit and Vegetables

For essential vitamins and minerals to boost your immune system

- We should include plenty of fruit and vegetables in our diets - aim to try and get five portions a day
- Your fruit and vegetables can be fresh, frozen, canned - they all count

Dairy Foods

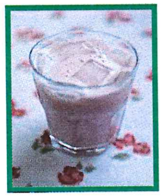

For strong bones

- People with COPD can have weaker bones. This can be a result of your medication or because you are less active
- Dairy foods include: cheese, milk, yoghurt, cream, fromage frais - include a helping or portion of dairy food in your diet three times per day

High Energy Foods

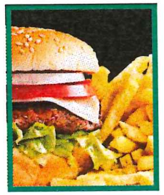

High energy foods are the ones to think about if you are trying to reduce your weight.

They tend to contain a lot of calories but don't provide many of the important nutrients for your body - try to cut back on these foods.

High energy foods are those that are high in fat (e.g. chips, fried foods, meat pies), high in sugar (e.g. fizzy drinks, sweets), or high in both sugar and fat (e.g. chocolate, cream cakes).

Diet and Breathing

If you become breathless when eating you may find it easier to eat three smaller meals and have snacks in between meals, however it is important not to reduce your overall food intake.

Eating and Smoking

Giving up smoking is a fantastic step to increasing your health and fitness but you may find your weight increases slightly. Don't be too concerned about this. The most important thing is to keep off cigarettes.

Eating and Exercise

If you are living with COPD it is very important to keep as active as possible, this helps your lungs and also the rest of your body to stay strong.

Try and gently increase the amount of activity you are doing yourself, and ask about local activity programmes.

Sponsored by an educational grant from Nutricia Ltd and supported by the Respiratory Dietitians Network
